# Can cycle day 7 FSH concentration during controlled ovarian stimulation be used to guide FSH dosing for in vitro fertilization?

**DOI:** 10.1186/1477-7827-11-12

**Published:** 2013-02-22

**Authors:** Yaakov Bentov, Eliezer Burstein, Courtney Firestone, Ross Firestone, Navid Esfandiari, Robert F Casper

**Affiliations:** 1 Toronto Centre for Advanced Reproductive Technology, Toronto, ON, Canada; 2 Division of Reproductive Sciences, Department of Obstetrics and Gynecology, University of Toronto, Toronto, ON, Canada; 3 Samuel Lunenfeld Research Institute, Mount Sinai Hospital, Toronto, ON, Canada

**Keywords:** Serum FSH concentration, FSH elimination, Poor ovarian response, In vitro fertilization, Ovarian stimulation

## Abstract

**Background:**

When stimulating a patient with poor ovarian response for IVF, the maximal dose of gonadotropins injected is often determined by arbitrary standards rather than a measured response. The purpose of this study was to determine if serum FSH concentration during an IVF stimulation cycle reflects follicular utilization of FSH and whether serum FSH values may inform dose adjustments of exogenous FSH.

**Methods:**

In this retrospective cross sectional study we studied 155 consecutive IVF cycles stimulated only with recombinant human FSH. We only included long GnRH agonist protocols in which endogenous FSH levels were suppressed. We correlated the serum concentration of cycle day (CD) 7 FSH with the number of oocytes retrieved, cleaving embryos and pregnancy rate.

**Results:**

We found that a CD7 FSH concentration above 22 IU/L was associated with poor response regardless of the daily dose of FSH injected and a lower pregnancy rate.

**Conclusions:**

We concluded that CD7 FSH concentration during stimulation could be used to guide FSH dosing in poor responders. If the CD7 FSH concentration is above 22 IU/L increasing the dose of FSH in an attempt to recruit more growing follicles is unlikely to be successful.

## Background

The stimulation of ovarian follicular development is an essential part of in vitro fertilization (IVF) and has a major impact on the treatment outcome. Over the years multiple protocols for ovarian stimulation have been developed containing different combinations of gonadotropins, GnRH analogues and other adjuvants [1-3]. Despite the differences between protocols it is clear that the major component responsible for follicular maturation is follicle stimulating hormone (FSH) [4]. Furthermore a direct dose response relationship between amount of FSH administered and follicle recruitment and growth has been described [5]. Typically, stimulation for IVF would involve daily subcutaneous injections of recombinant or urinary-derived FSH usually at a constant daily dose for 10–13 days.

Poor ovarian response may be defined as a low oocyte yield despite treatment with a high dose of FSH [6]. Poor ovarian response is one of the main obstacles to pregnancy success in IVF since treatment is dependent on the quantity as well as the quality of oocytes and embryos. Since poor ovarian response involves resistance to the action of FSH, one way to improve response is to increase the daily dose of FSH administered. This practice potentially allows follicles with fewer FSH receptors, and requiring a higher FSH threshold, to respond to the stimulation. However, since the only parameter currently used to adjust the dose of FSH is ovarian response, and since injectable FSH preparations are still quite expensive, it would be valuable to have an early prospective marker of whether increasing the dose of FSH would be useful or not. Also, the concept of “the maximal” daily dose of FSH given to a poor responding patient is dependent mostly on clinic policy without any objective tools to guide the decision.

Factors other than the daily dose of FSH may affect the serum concentration of FSH. The measured serum concentration represents the balance between the rate of absorption and the rate of elimination of FSH. With consecutive daily administration of FSH, steady state levels are thought to be reached after 3–5 days of treatment [7]. Endogenous FSH and exogenous FSH with altered patterns of glycosylation have different rates of elimination [8]. Urinary derived FSH contains a higher proportion of acidic isoforms and as a result is characterized by a slower clearance rate and lower bioactivity relative to recombinant FSH [8]. Moreover, stimulation cycles that include down regulation of the pituitary secretion of endogenous FSH show an increased exogenous FSH Cmax [5]. Another mechanism by which FSH is cleared from the circulation involves binding to its receptor [9]. This could explain why the more bioactive form of FSH is also cleared faster [8]. We speculated that women with a poor ovarian response would have a lower number of follicles that may contain few FSH receptors, and as a result would show a lower rate of FSH clearance and a higher steady state serum FSH concentration compared to good responders. We hypothesized that monitoring the serum concentration of FSH during ovarian stimulation could provide information on whether exogenous FSH is being utilized efficiently. The objective of the present study was to correlate serum FSH concentrations in the early phase of ovarian stimulation with the number of oocytes retrieved, embryo development and pregnancy outcome.

## Methods

To test our hypothesis, we conducted a retrospective cross-sectional study that included all IVF cycles conducted at the Toronto Center for Advanced Reproductive Technology (TCART) between January 2008 and May 2012 that met the following inclusion criteria.

•Long mid luteal GnRH agonist down regulation protocol

•Only recombinant FSH was used for ovarian stimulation

•The daily dose of recFSH was 200–300 international units a day.

Exclusion criteria were:

•Cycles that were cancelled prior to oocyte retrieval

•Missing data on baseline FSH concentration and on cycle day 7 as well as the number of oocytes, embryos or outcome.

•Incomplete down regulation (CD3 FSH ≥ 10 or CD3 Estradiol ≥ 200 pmol/L)

The search of patient data was approved by the Mount Sinai Hospital institutional ethics committee. FSH used by patients in the study was recombinant FSH (Gonal-F® Serono, Oakville, Ontario, Canada or Puregon®, Merck, Scarborough, Ontario, Canada). We administered 10,000 IU of hCG as a single subcutaneous injection (human chorionic gonadotropin, Pregnyl®, Merck, Scarborough, Ontario, Canada) 36 hours before egg retrieval when at least two follicles measured 18 mm in diameter. All cycles received luteal phase support with progesterone in the form of vaginal suppositories (Kingsway Drugs, Toronto, Ontario, Canada) 200 mg three times daily. The long luteal protocol involved downregulation with a GnRH agonist, buserelin acetate (Suprefact, Sanofi-Aventis Canada Inc) started about one week before menses and stimulation with recFSH was started on day 3 (CD3) of the next cycle and continued until the administration of the hCG trigger injection. In these cycles patients were seen on cycle day 3 for a blood test (estradiol and FSH) and ultrasound and again on cycle day 7 (CD7). Between these days the patient was self-administering the medication and no changes were made to dose.

Analysis of FSH serum concentration was done using the Vitros ECiQ Immunodiagnostic System (Ortho-Clinical diagnostics, Johnson and Johnson, Rochester, NY 14626–5101). The analytical sensitivity of this assay is typically 0.50 IU/L. The intra-assay variability was 1.7-2.8% and inter-assay variability was 3.7-10.6%.

Statistical analysis was done using GraphPad statistical analyzer. Due to a non-normal distribution, non-parametric tests were used (Kruskal-Wallis test) to test the significance of continuous parameters and Fisher’s exact test for binomial parameters. The correlation between serum FSH on cycle day 7 and the dose of FSH administered was compared using one way ANOVA test. A p-value of ≤0.05 was considered as significant.

## Results

We were able to identify 155 cycles within the period of time between January 2008 and May 2012 that met all of the inclusion and exclusion criteria.

**Figure 1:**
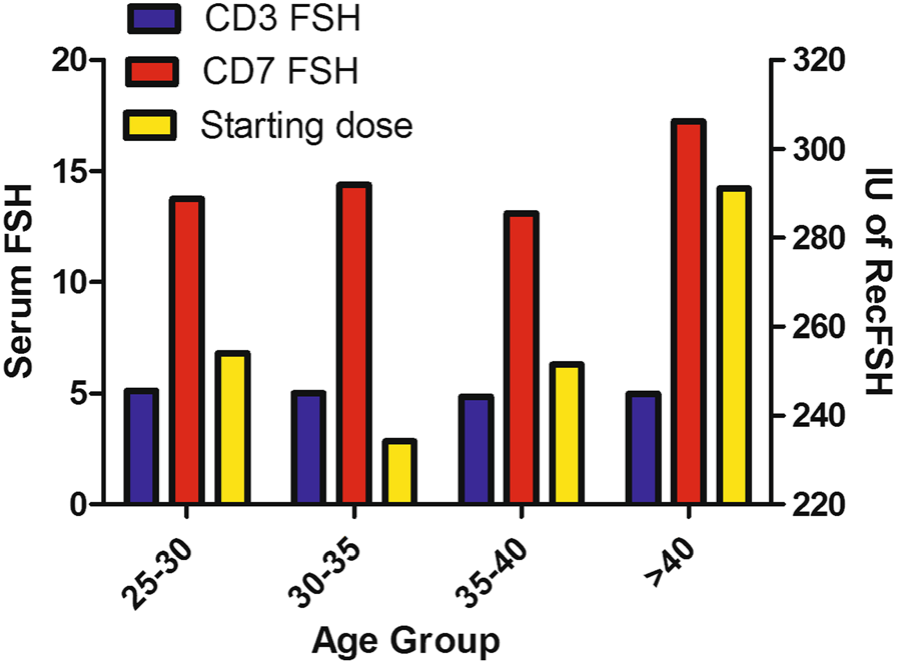
**CD3 & CD7 FSH and recFSH dose.** Serum FSH concentration on cycle day 3 and 7 (primary Y axis) and average starting daily dose of recFSH (secondary Y axis) in the different age groups.

Patient characteristics are detailed in Table 1. The average age of patients included in the study was 35.8 years (21–45), Average BMI was 24.5 (17–41) and baseline CD3 FSH was 6.5 (2.4-20) prior to the IVF cycle. Late maternal age (37 and older) accounted for 44.5% of the patients; male factor infertility was associated with 56% of the cycles and tubal factor in 23.1% (Table 1). All cycles included in the study were of the long mid luteal GnRH agonist down-regulation protocol with recFSH only used for ovarian stimulation. The average daily dose of recFSH administered was 250.3 IU (200–300 IU). The increase in dose of recFSH is typically done in increments of 25 IU and therefore was not regarded as a continuous variable. The distribution of the different starting doses of RecFSH is presented in Table 2. The serum concentration of CD7 FSH for the 4 daily FSH dose groups were 11.35, 10.23, 4.96, and 13.88 IU/L respectively (Figure 1 and Table 2). With GnRH agonist downregulation there was no difference in day 3 FSH concentrations in the stimulation cycles (Figure 1). Despite the trend for an increased serum FSH with a higher dose of injected FSH the differences using one way Anova was not significant (pValue = 0.0502).

**Table 1:** Patient characteristics

		**Average**	**Median**	**Range**	**Standard deviation**	**SEM**
*Age*		35.8	36	21-45	3.61	0.294
*BMI*		24.5	23.5	17-41	4.78	0.74
*Baseline CD3 FSH*		6.5	6	2.4-20	3.08	0.469
**Diagnostic groups**						
*Late maternal age (>36.9)*	44.5%					
*Tubal Factor*	23.1%					
*Tubal + Hydroslpings*	0.0%					
*Non-Ovulatory*	1.1%					
*Male factor*	56.0%					
*Endometriosis stage 1 + 2*	2.2%					
*Endometriosis stage 3 + 4*	6.6%					
*Unexplained*	8.8%					
*Repeated pregnancy loss*	1.1%					

Characteristics of the patients included in the study. For each parameter we describe, the average, median, range, standard deviation, number of observations and the standard error of the mean.

**Table 2:** Distribution of starting daily dose of RecFSH

**Starting daily FSH dose**	**Percentage of patients**	**CD7 Serum FSH (IU/L)**
200	35.9%	11.35
225	9.0%	10.23
250	14.7%	14.96
275	0.0%	
300	40.4%	13.88

The distribution of the different starting doses of RecFSH and their corresponding CD7 serum FSH.

**Figure 2:**
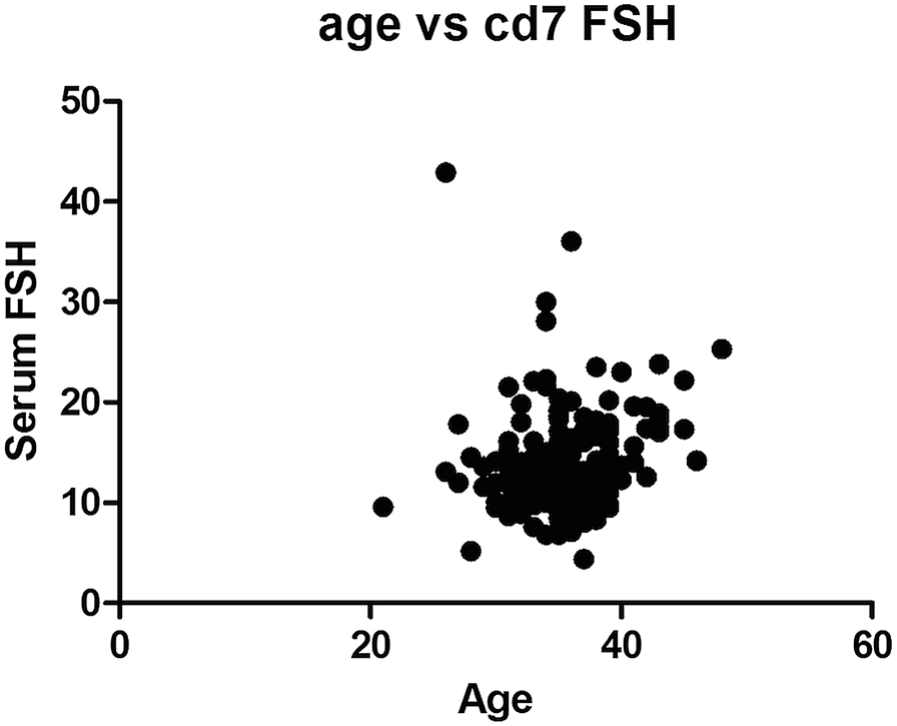
**CD7 serum FSH in relation to age.** XY graph showing serum FSH concentration on CD7 according to the patient’s age.

The correlation between the age of the patients and CD7 serum FSH was analyzed with Pearson’s correlation and was found to be non-significant (R square 0.016 and p Value = 0.089) (Figure 2). Cycle characteristics are described in Table 3.

**Table 3:** IVF cycle characteristics

	**Average**	**Median**	**Range**	**Standard deviation**	**SEM**
*CD3 FSH (IU/L)*	5.0	4.9	0.15-9.5	1.53	0.131
*CD3 Estradiol (pmol/L)*	98.3	92.4	3-200	39.9	3.31
*CD3 LH (IU/L)*	3.8	3.4	0.1-12.2	2.45	0.21
*Dose of FSH (IU)*	249.6	250	200-300	44.05	3.12
*Day of HCG*	12.7	13	9-18	1.51	0.12
*Estradiol on day of hCG (pmol/L)*	9,862.2	8,639	567.8-36,571	7,066.3	624.6

The characteristics of all cycles included in the study (CD3 – cycle day 3).

**Figure 3:**
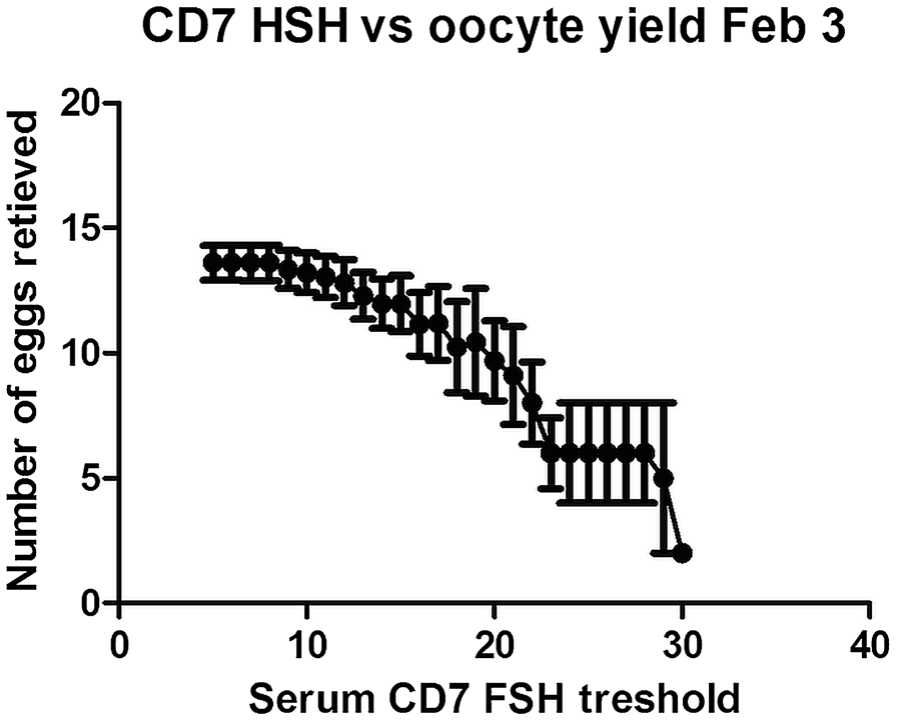
**CD7 FSH and oocyte yield.** Oocyte yields with different thresholds of serum FSH on CD7 to define a high level starting with a threshold of > 5 IU/L until 30 IU/L. Error bars indicate the standard error of the mean.

Figure 3 describes the correlation between different thresholds of serum FSH on cycle day 7 and the corresponding oocyte yield. The thresholds examined start with a serum FSH concentration of more than 5 IU/L and up to > 30 IU/L. The average oocyte yield was 13.7 for the lowest threshold and 2 for the highest. Correlation analysis using the Spearman analysis was significant with a p Value <0.0001 (r = −0.9863).

**Figure 4:**
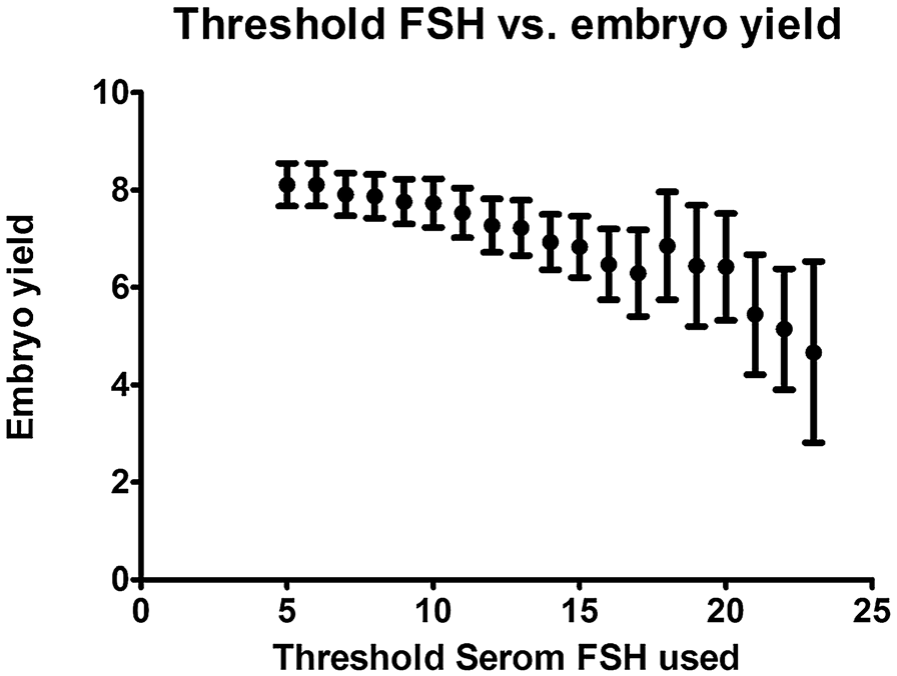
**CD7 FSH and embryo yield.** Embryo yields with different thresholds of serum FSH on CD7 to define a high level starting with a threshold of > 5 IU/L until 30 IU/L. Error bars indicate the standard error of the mean.

Figure 4 describes the correlation between CD7 serum FSH concentration and the yield of embryos. Here we used the same thresholds of more than 5 and up to >30 IU/L. Mean embryo yield for the lowest threshold was 8.11 and zero for the highest. Spearman analysis showed a significant correlation with a p Value <0.0001 (r = −0.9803).

**Figure 5:**
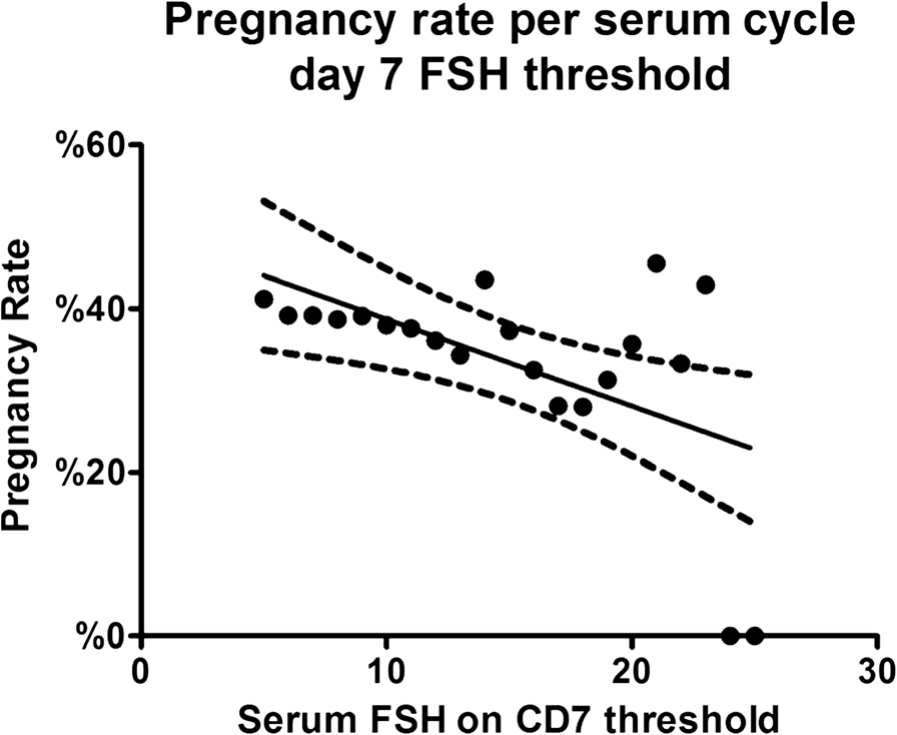
**CD7 FSH and pregnancy rate.** Pregnancy rates with different thresholds of serum FSH on CD7 to define a high level starting with a threshold of > 5 IU/L until 30 IU/L. The figure also describes the linear regression line as well as the 95% confidence interval range.

Figure 5 describes the association of pregnancy rate with different thresholds of CD7 FSH. The pregnancy rate for a CD7 FSH > 5 was 41.2% and went down to zero with a CD7 FSH threshold > 23 IU/L. The figure also shows the linear regression line and the 95% confidence interval range. R square for goodness of fit is 0.3016 and the p value <0.01.

## Discussion

Unlike many expensive medical treatments that are covered by health insurance, infertility treatment is usually paid for out of pocket. A recent publication estimated the median cost of an IVF cycle in the United States at $24,373, with the cost of an unsuccessful IVF treatment even higher ($25,921) [10]. A significant portion of the total cost of IVF is spent on gonadotropin injections. This observation is especially true in poor responders who are usually treated with high daily doses of FSH, at times exceeding 600 IU a day. Unfortunately, after several days of injections and a few thousand dollars spent on fertility drugs, the cycle may be cancelled for lack of adequate response, or oocyte retrieval will be pursued with less than the desired yield of oocytes.

In the present study, we show that a significantly higher concentration of serum FSH following administration of exogenous FSH did not result in a higher number of oocytes and embryos. The administration of similar doses of FSH resulted in a wide range of serum FSH concentrations (6.8-36 IU/L) on CD 7 that negatively correlated with oocyte yields. The steady state of any substance in the serum represents its rate of intake and clearance. In our clinic we see the patients on CD3 when they start gonadotropin administration and again on day 7. In between these days the patients are not monitored and no changes are made to the dose of gonadotropins. These five days between the start of gonadotropin injections and the next measurement of its serum concentration is the time in which injected gonadotropins were shown to reach a steady state. Despite the lack of significance, it is clear that there is a correlation between the injected dose of FSH and its serum concentration. However, the narrow range of daily FSH dose,(200–300 IU a day) was associated with a much wider range of serum FSH concentrations, with the highest concentrations being 6 fold greater than the lowest. This observation suggests that the rate of FSH clearance plays an important role in its serum concentration. This study is retrospective and, therefore, possibly subject to unrecognized biases. In order to reduce the impact of age on endogenous FSH concentrations, we only included cycles with a long mid luteal GnRH agonist protocol in order to reduce the contribution of endogenous FSH as much as possible. As seen in Figure 1, the cycle day 3 FSH concentration was similar in all 4 age groups (average of 5 IU/L) after pituitary downregulation by GnRH agonist. Moreover, only cycles with recFSH were included in order to prevent differences in the rate of clearance that are attributed to difference in acidic isoforms between recFSH and urinary gonadotropins [8]. Although long luteal protocols are considered to be overly suppressive for some older patients, we felt that the use of this protocol was the only way to eliminate the endogenous FSH differences between groups that would preclude any meaningful interpretation of the data.

In our study, serum concentration of FSH on CD7 showed a significant negative correlation with oocyte and embryo yield, as well as pregnancy rates despite the administration of a similar dose of FSH. The changes in serum FSH concentration on CD7 cannot be attributed to the baseline FSH or to the patient’s age since both did not show a significant correlation.

At least part of the process of clearance of FSH from the circulation involves binding of FSH to its receptor and internalization of the hormone receptor complex. Therefore, a low number of follicles containing FSH receptors on granulosa cells will inevitably result in the accumulation of FSH in circulation as a result of less binding of FSH to FSHR [9].

We observed that the serum FSH concentration on CD7 predicted response to FSH administration with a dramatic change in oocyte retrieval numbers and pregnancy outcome above a serum FSH concentration of 22 IU/L. Our results demonstrate that there was excessive circulating FSH in poor responders and suggest that increasing the dose of FSH would not be helpful since the FSH administered already was not being completely used. This observation is consistent with our clinical experience that doses of FSH above 300 IU per day are unlikely to increase follicular response in older women or previous poor responders, and only adjunctive measures are likely to be of help in increasing oocyte yield. These include sensitizing the follicles using androgens prior to ovarian stimulation [11] or by adding LH or hCG to recFSH during ovarian stimulation [12]. Alternatively, if the FSH serum concentration on CD7 is less than 22 IU/L, there may be a place for increasing FSH dose if follicular response is less than desired.

## Conclusions

In conclusion, the only parameter currently used to guide the choice of FSH dosing is the response of the ovary, i.e. follicular development and estrogen production. However, with poor response, it is difficult to decide whether the dose of FSH should be increased. Using a cut-off of 22 IU/L of serum FSH on CD7 may provide a simple tool to guide this decision.

## Abbreviations

CD: Cycle day; FSH: Follicular stimulating hormone; FSHR: FSH receptor; GnRH: Gonadotropin realising hormone; hCH: Human chorionic gonadotropin; ICSI: Intra-cytoplasmic injection; IU: International units; IVF: In vitro fertilization; LH: Luteinizing hormone; OHSS: Ovarian hyper-stimulation syndrome; recFSH: Recombinant FSH.

## Competing interests

The authors declare that they have no competing interests.

## Authors’ contribution

YB collected, analyzed the data and wrote the manuscript. EB, CF and RS collected the data, RFC and NE supervised the study and revised the manuscript. All authors read and approved the final revision of manuscript.
